# Crystal structure of CsCrAs_2_O_7_, a new member of the diarsenate family

**DOI:** 10.1107/S205698901500910X

**Published:** 2015-05-16

**Authors:** Mohamad Alem Bouhassine, Habib Boughzala

**Affiliations:** a Laboratoire de Materiaux et Cristallochimie, Faculté des Sciences de Tunis, Université de Tunis El Manar, 2092 Manar II Tunis, Tunisia

**Keywords:** crystal structure, isotypism, chromium, caesium, diarsenate, channel structure

## Abstract

Caesium chromium(III) diarsenate(V), CsCrAs_2_O_7_, was prepared by solid-state reactions. The title structure consists of isolated CrO_6_ octa­hedra and As_2_O_7_ diarsenate groups, sharing corners to build up a three-dimensional [CrAs_2_O_7_]^−^ anionic framework. In this framework, channels extending parallel to [001] are present in which the ten-coordinate Cs^+^ ions reside. CsCrAs_2_O_7_ is isotypic with the monoclinic *A* ^I^ *M* ^III^ *X*_2_O_7_ (*A* ^I^ = alkali metal; *M* ^III^ = Al, Cr, Fe; *X* = As, P) type I family of compounds crystallizing in the space group *P*2_1_/*c*.

## Abstract 2

The structure of CsCrAs_2_O_7_ can be described as a three-dimensional [CrAs_2_O_7_]^−^ anionic framework in which the Cs^+^ cations are located in empty channels running along [001].

## Chemical context   

In recent years, inorganic metal phosphates and arsenates with formula *A* ^I^ *M* ^III^ *X*_2_O_7_ (*A* ^I^ = alkali metal; *M* ^III^ = Al, Cr, Fe; *X* = As, P) have been part of intensive research activities, with crystals grown either from high-temperature solid-state reactions or under aqueous solution conditions. The crystal chemistry of these compounds with *X*_2_O_7_ groups reveals a large structural variety accompanied in some cases by inter­esting magnetic, electric, optical, or thermal expansion properties. Focusing on compounds with *M* ^III^ = Cr, it is noticeable that corresponding diphosphates have been studied extensively, in contrast to the scarcely studied chromium diarsen­ates. Herein the preparation and crystal structure of CsCrAs_2_O_7_ is reported, one of a series of new cesium chromium(III) arsenate compounds recently isolated by our group.

## Structural commentary   

**Figure 1:**
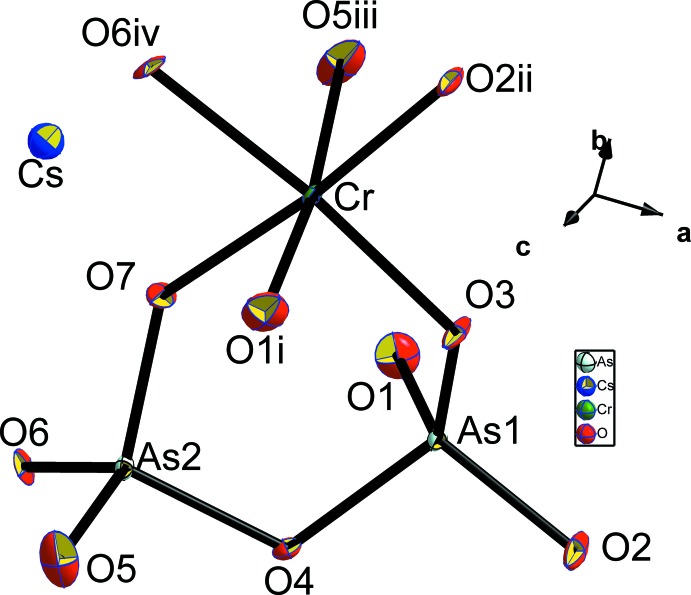
The coordination polyhedra around Cr and As atoms in the title structure. Displacement ellipsoids are drawn at the 50% probability level. [Symmetry codes: (i) *x*, 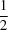 − *y*, 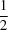 + *z*; (ii) 2 − *x*, 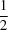 + *y*, 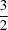 − *z*; (iii) *x*, 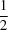 − *y*, −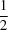 + *z*; (iv) 1 − *x*, 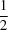 + *y*, 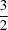 − *z*.]

**Figure 2:**
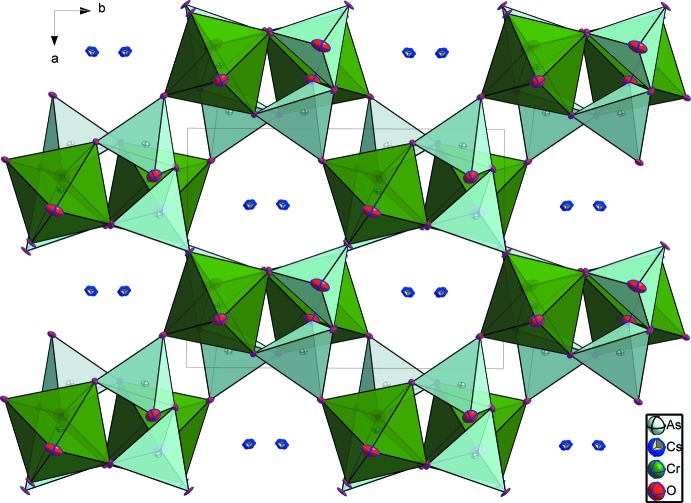
Projection of the CsCrAs_2_O_7_ structure showing the channels parallel to [001] in which the Cs^+^ cations are located.

The structure of CsCrAs_2_O_7_ can be described as a three-dimensional [CrAs_2_O_7_]^−^ anionic framework (Fig. 1 ▸) with channels extending parallel to [001] that are occupied by ten-coordinate Cs^+^ cations (Fig. 2 ▸).

**Figure 3:**
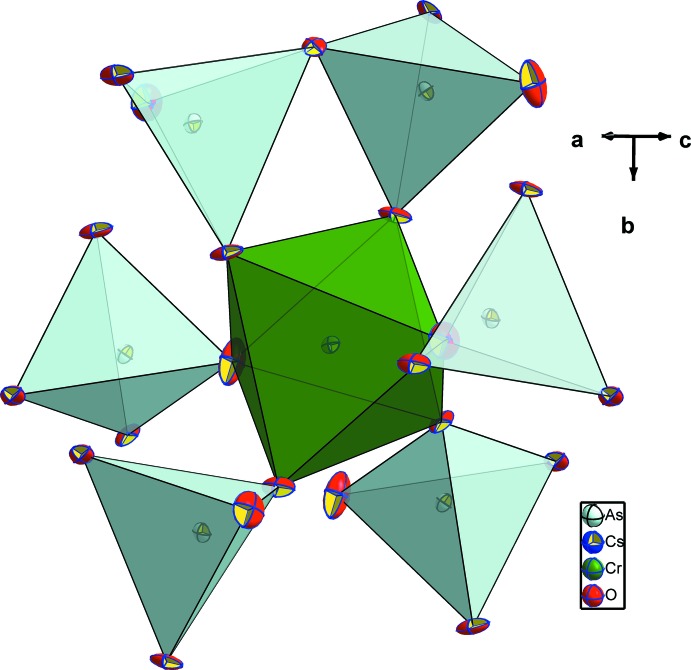
The environment of the CrO_6_ octa­hedron in the structure of CsCrAs_2_O_7_.

**Figure 4:**
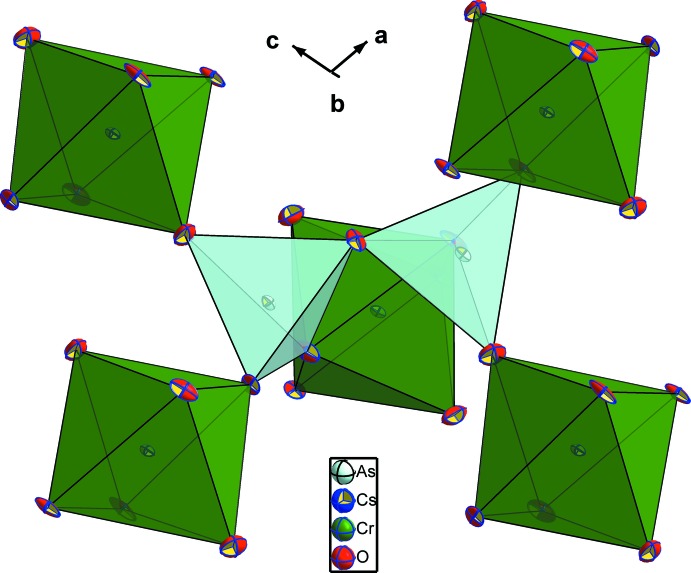
The environment of the diarsenate group in the structure of CsCrAs_2_O_7_.

**Figure 5:**
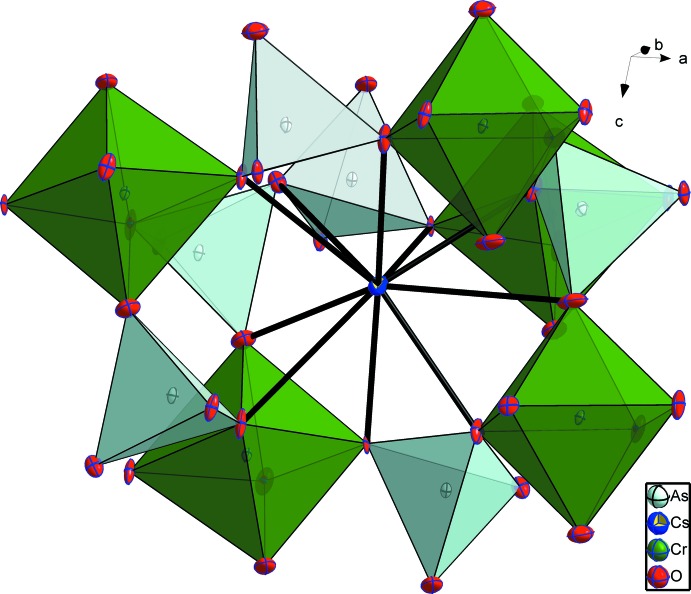
The surrounding of the ten-coordinated Cs^+^ cation in the structure of CsCrAs_2_O_7_.

The two independent arsenic atoms form AsO_4_ tetra­hedra and are connected *via* the bridging O4 atom into a diarsenate As_2_O_7_ anion. Like in the related structures of KAlAs_2_O_7_ (Boughzala & Jouini, 1995 ▸) and RbAlAs_2_O_7_ (Boughzala *et al.*, 1993 ▸), the As—O distances involving the bridging O4 atom are the longest (Table 1 ▸). The As1—O4—As2 bridging angle of 118.7 (2)° in the title structure is somewhat smaller than that of 125.9 (2)° reported for the isotypic structure of CsCrP_2_O_7_ (Linde & Gorbunova, 1982 ▸). The O—As—O bond angles span a range between 103.8 (2) and 116.2 (2)° and 105.5 (2) and 115.6 (2)°, respectively, for As1 and As2, reflecting the distortion of each of the AsO_4_ tetra­hedra. The Cr^III^ cations are in a slightly distorted octa­hedral oxygen coordination with Cr—O distances ranging from 1.944 (4) to 2.010 (4) Å (Table 1 ▸), and with O—Cr—O angles ranging from 82.96 (18) to 95.94 (17)° and from 172.37 (19) to 173.72 (17)°. Each CrO_6_ octa­hedron shares its corners with five As_2_O_7_ anions, one of which is chelating and the others belonging to four different As_2_O_7_ groups (Fig. 3 ▸). On the other hand, each As_2_O_7_ anion is surrounded by five CrO_6_ octa­hedra as depicted in Fig. 4 ▸. The environment of the ten-coordinate Cs^+^ cation situated in the cavities of the resulting [CrAs_2_O_7_]^−^ framework is shown in Fig. 5 ▸.

**Figure 6:**
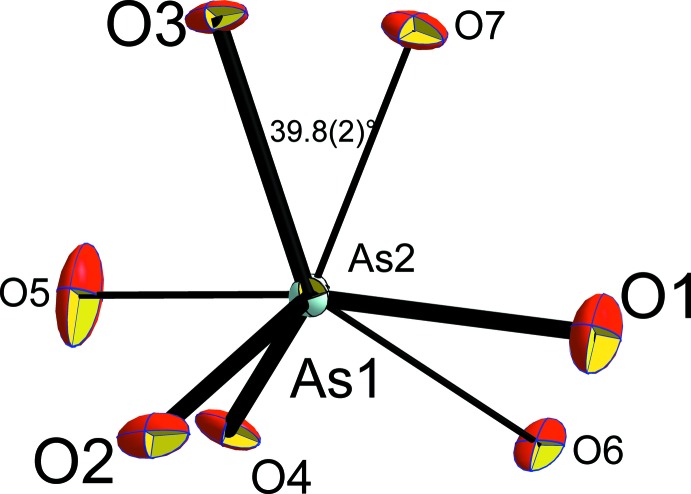
View parallel to the As1—As2 direction, emphasizing the nearly eclipsed conformation of the diarsenate anion.

It is worth mentioning that in the related aluminium diarsenate family *A* ^I^AlAs_2_O_7_ (*A* ^I^= K, Rb, Tl, Cs) (Boughzala & Jouini, 1992 ▸) that crystallizes isotypically in space group *P* 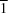 and is classified as type II, the diarsenate groups have a different conformational orientation as those of the title structure. In the title structure, belonging to the type I family of *A* ^I^ *M* ^III^ *X*_2_O_7_ diarsenates, the diarsenate tetra­hedra are in a nearly eclipsed conformation with a torsion angle O3—As1—As2—O7 of 39.8 (2)°, as shown in Fig. 6 ▸. The corresponding angle is 158.8 (2)° for KAlAs_2_O_7_ (Boughzala & Jouini, 1995 ▸).

Using the bond-valence method (Brown, 2002 ▸), the calculated bond-valence-sum values (in valence units) of 5.08, 4.97, 3.01 and 1.35, respectively, for As1, As2, Cr and Cs are in good agreement with the expected oxidation states.

## Database survey   

The structure of KAlP_2_O_7_ (Ng & Calvo, 1973 ▸) was the first published of the *A* ^I^ *M* ^III^ *X*_2_O_7_ family. Afterwards, based on different substitutions and combinations, a large number of different phases were isolated and crystallographically characterized. Replacement of one of the cations can improve the structural and physical properties but also affects the coordin­ation numbers, the degree of distortion of the coord­ination polyhedra and the conformation of the *X*_2_O_7_ groups. Also, the crystal symmetry can be affected. The structures are triclinic, in space group *P* 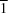 with two formulas units, for the diarsenate compounds *A* ^I^AlAs_2_O_7_ (*A* ^I^= K, Rb, Tl, Cs) (Boughzala & Jouini, 1992 ▸; Boughzala *et al.*, 1993 ▸; Boughzala & Jouini; 1995 ▸), whereas diphosphates are generally monoclinic. The isotypic *A* ^I^CrP_2_O_7_ phases crystallize in space group *P*2_1_/*c* for *A* ^I^ = Na (Bohaty *et al.*, 1982 ▸), K (Gentil *et al.*, 1997 ▸), Rb (Zhao & Li, 2011 ▸) and Cs (Linde & Gorbunova, 1982 ▸). The same counts for the *A* ^I^FeP_2_O_7_ phases for *A* ^I^ = Na (Gabelica-Robert *et al.*, 1982 ▸) and K (Riou *et al.*, 1988 ▸). However, the two Li-containing phases Li*M*P_2_O_7_ show a symmetry reduction to space group *P*2_1_ (*M* = Cr, Ivashkevich *et al.*, 2007 ▸; *M* = Fe, Riou *et al.*, 1990 ▸).

## Synthesis and crystallization   

The crystals of the title compound were obtained from heating a mixture of Cs_2_CO_3_, Cr_2_O_3_ and NH_4_H_2_AsO_4_, with a Cs:Cr:As molar ratio of 1:1:2. In order to eliminate volatile products, the sample was placed in a porcelain crucible and slowly heated under atmospheric conditions to 673 K and kept at that temperature for 24 h. In a second step, the crucible was progressively heated at 1023 K for 4 days and then slowly cooled down at a rate of 5 K/24 h to 923 K and finally quenched to room temperature. The product was washed with water and rinsed with an aqueous solution of HCl. Two phases could be isolated. The major phase forms regular cube-shaped dark-green crystals of yet unknown composition. The second phase represents the title compound and was obtained in the form of pink crystals.

## Refinement   

Crystal data, data collection and structure refinement details are summarized in Table 2 ▸. The maximum and minimum electron density in the final difference Fourier map is located at 0.95 Å, 0.87 Å, respectively, from the Cs atom.

## Supplementary Material

Crystal structure: contains datablock(s) I. DOI: 10.1107/S205698901500910X/wm5157sup1.cif

Structure factors: contains datablock(s) I. DOI: 10.1107/S205698901500910X/wm5157Isup2.hkl

CCDC reference: 1400446

Additional supporting information:  crystallographic information; 3D view; checkCIF report

## Figures and Tables

**Table 1:** Selected bond lengths ()

CrO5^i^	1.944(4)	As1O2	1.664(4)
CrO7	1.954(4)	As1O3	1.681(4)
CrO1^ii^	1.978(4)	As1O4	1.763(4)
CrO3	1.982(4)	As2O5	1.641(4)
CrO2^iii^	2.007(4)	As2O6	1.661(4)
CrO6^iv^	2.010(4)	As2O7	1.669(4)
As1O1	1.651(4)	As2O4	1.750(4)

Symmetry codes: (i) 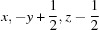; (ii) 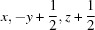; (iii) 
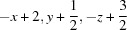
; (iv) 
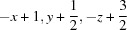
.

**Table 2:** Experimental details

Crystal data	
Chemical formula	CsCrAs_2_O_7_
*M*_r_	446.75
Crystal system, space group	Monoclinic, *P*2_1_/*c*
Temperature (K)	293
*a*, *b*, *c* ()	7.908(1), 10.0806(10), 8.6371(10)
()	105.841(1)
*V* (^3^)	662.38(13)
*Z*	4
Radiation type	Mo *K*
(mm^1^)	17.05
Crystal size (mm)	0.20 0.20 0.10

Data collection	
Diffractometer	EnrafNonius CAD-4
Absorption correction	scan (North *et al.*, 1968 ▸)
*T*_min_, *T*_max_	0.132, 0.281
No. of measured, independent and observed [*I* > 2(*I*)] reflections	1530, 1433, 1205
*R*_int_	0.051
(sin /)_max_ (^1^)	0.637

Refinement	
*R*[*F* ^2^ > 2(*F* ^2^)], *wR*(*F* ^2^), *S*	0.027, 0.075, 1.13
No. of reflections	1433
No. of parameters	101
_max_, _min_ (e ^3^)	1.60, 1.23

Computer programs: *CAD-4 EXPRESS* (EnrafNonius, 1994 ▸), *XCAD4* (Harms Wocadlo, 1995 ▸), *SHELXS97* and *SHELXL97* (Sheldrick, 2008 ▸), *DIAMOND* (Brandenburg, 2008 ▸) and *publCIF* (Westrip, 2010 ▸).

## supplementary crystallographic information

### Crystal data

**Table:**

CsCrAs_2_O_7_	*F*(000) = 804
*M**_r_* = 446.75	*D*_x_ = 4.480 Mg m^−^^3^
Monoclinic, *P*2_1_/*c*	Mo *K*α radiation, λ = 0.71073 Å
Hall symbol: -P 2ybc	Cell parameters from 25 reflections
*a* = 7.908 (1) Å	θ = 3.8–27°
*b* = 10.0806 (10) Å	µ = 17.05 mm^−^^1^
*c* = 8.6371 (10) Å	*T* = 293 K
β = 105.841 (1)°	Monoclinic, pink
*V* = 662.38 (13) Å^3^	0.20 × 0.20 × 0.10 mm
*Z* = 4	

### Data collection

**Table:**

Enraf–Nonius CAD-4 diffractometer	1205 reflections with *I* > 2σ(*I*)
Radiation source: fine-focus sealed tube	*R*_int_ = 0.051
Graphite monochromator	θ_max_ = 26.9°, θ_min_ = 2.7°
ω/2θ scans	*h* = −10→9
Absorption correction: ψ scan (North *et al.*, 1968)	*k* = 0→12
*T*_min_ = 0.132, *T*_max_ = 0.281	*l* = 0→11
1530 measured reflections	2 standard reflections every 120 min
1433 independent reflections	intensity decay: 1.1%

### Refinement

**Table:**

Refinement on *F*^2^	Primary atom site location: structure-invariant direct methods
Least-squares matrix: full	Secondary atom site location: difference Fourier map
*R*[*F*^2^ > 2σ(*F*^2^)] = 0.027	*w* = 1/[σ^2^(*F*_o_^2^) + (0.043*P*)^2^ + 1.1495*P*] where *P* = (*F*_o_^2^ + 2*F*_c_^2^)/3
*wR*(*F*^2^) = 0.075	(Δ/σ)_max_ < 0.001
*S* = 1.13	Δρ_max_ = 1.60 e Å^−^^3^
1433 reflections	Δρ_min_ = −1.23 e Å^−^^3^
101 parameters	Extinction correction: *SHELXL97* (Sheldrick, 2008), Fc^*^=kFc[1+0.001xFc^2^λ^3^/sin(2θ)]^-1/4^
0 restraints	Extinction coefficient: 0.0018 (4)

### Special details

**Table:**

Geometry. All e.s.d.'s (except the e.s.d. in the dihedral angle between two l.s. planes) are estimated using the full covariance matrix. The cell e.s.d.'s are taken into account individually in the estimation of e.s.d.'s in distances, angles and torsion angles; correlations between e.s.d.'s in cell parameters are only used when they are defined by crystal symmetry. An approximate (isotropic) treatment of cell e.s.d.'s is used for estimating e.s.d.'s involving l.s. planes.
Refinement. Refinement of *F*^2^ against ALL reflections. The weighted *R*-factor *wR* and goodness of fit *S* are based on *F*^2^, conventional *R*-factors *R* are based on *F*, with *F* set to zero for negative *F*^2^. The threshold expression of *F*^2^ > σ(*F*^2^) is used only for calculating *R*-factors(gt) *etc*. and is not relevant to the choice of reflections for refinement. *R*-factors based on *F*^2^ are statistically about twice as large as those based on *F*, and *R*- factors based on ALL data will be even larger.

### Fractional atomic coordinates and isotropic or equivalent isotropic displacement parameters (Å^2^)

**Table:**

	*x*	*y*	*z*	*U*_iso_*/*U*_eq_	
As1	0.93130 (7)	0.13138 (6)	0.68578 (6)	0.00527 (16)	
As2	0.63567 (7)	0.08924 (5)	0.84021 (6)	0.00495 (16)	
Cs	0.31839 (5)	0.19797 (4)	0.45751 (4)	0.01429 (15)	
Cr	0.73911 (11)	0.39967 (8)	0.76602 (10)	0.0043 (2)	
O1	0.8001 (6)	0.1049 (5)	0.5039 (5)	0.0168 (10)	
O2	1.1355 (5)	0.0742 (4)	0.7202 (5)	0.0122 (9)	
O3	0.9413 (5)	0.2889 (4)	0.7514 (5)	0.0099 (8)	
O4	0.8363 (5)	0.0371 (4)	0.8122 (5)	0.0093 (8)	
O5	0.6538 (6)	0.0802 (5)	1.0338 (5)	0.0199 (10)	
O6	0.4833 (5)	−0.0082 (4)	0.7244 (5)	0.0085 (8)	
O7	0.5967 (5)	0.2403 (4)	0.7594 (5)	0.0109 (8)	

### Atomic displacement parameters (Å^2^)

**Table:**

	*U*^11^	*U*^22^	*U*^33^	*U*^12^	*U*^13^	*U*^23^
As1	0.0033 (3)	0.0057 (3)	0.0069 (3)	0.0003 (2)	0.0014 (2)	−0.0003 (2)
As2	0.0034 (3)	0.0055 (3)	0.0059 (3)	−0.0009 (2)	0.0013 (2)	−0.0002 (2)
Cs	0.0106 (2)	0.0165 (2)	0.0136 (2)	−0.00201 (14)	−0.00033 (14)	0.00299 (14)
Cr	0.0026 (4)	0.0045 (4)	0.0055 (4)	0.0004 (3)	0.0005 (3)	−0.0002 (3)
O1	0.017 (2)	0.022 (2)	0.010 (2)	−0.0029 (19)	0.0010 (17)	−0.0022 (18)
O2	0.0044 (19)	0.007 (2)	0.025 (2)	−0.0020 (16)	0.0035 (16)	−0.0024 (17)
O3	0.0040 (18)	0.0045 (19)	0.020 (2)	0.0006 (15)	0.0012 (16)	−0.0032 (16)
O4	0.0077 (19)	0.0067 (19)	0.015 (2)	0.0038 (15)	0.0049 (15)	0.0035 (16)
O5	0.016 (2)	0.035 (3)	0.007 (2)	−0.009 (2)	0.0016 (17)	−0.0009 (18)
O6	0.0065 (19)	0.008 (2)	0.011 (2)	−0.0061 (16)	0.0024 (15)	−0.0007 (16)
O7	0.0074 (19)	0.0047 (19)	0.020 (2)	−0.0012 (16)	0.0018 (15)	0.0030 (17)

### Geometric parameters (Å, º)

**Table:**

Cs—O7	2.948 (4)	Cr—O1^v^	1.978 (4)
Cs—O3^i^	3.030 (4)	Cr—O3	1.982 (4)
Cs—O6	3.113 (4)	Cr—O2^vi^	2.007 (4)
Cs—O6^ii^	3.152 (4)	Cr—O6^vii^	2.010 (4)
Cs—O2^i^	3.155 (4)	As1—O1	1.651 (4)
Cs—O7^iii^	3.196 (4)	As1—O2	1.664 (4)
Cs—O1^ii^	3.237 (5)	As1—O3	1.681 (4)
Cs—O2^iv^	3.253 (4)	As1—O4	1.763 (4)
Cs—O4^ii^	3.314 (4)	As2—O5	1.641 (4)
Cs—O5^iii^	3.393 (5)	As2—O6	1.661 (4)
Cr—O5^iii^	1.944 (4)	As2—O7	1.669 (4)
Cr—O7	1.954 (4)	As2—O4	1.750 (4)
			
O1—As1—O2	116.2 (2)	O6^ii^—Cs—O5^iii^	91.60 (11)
O1—As1—O3	115.7 (2)	O2^i^—Cs—O5^iii^	80.89 (11)
O2—As1—O3	108.3 (2)	O7^iii^—Cs—O5^iii^	50.19 (10)
O1—As1—O4	103.8 (2)	O1^ii^—Cs—O5^iii^	146.54 (11)
O2—As1—O4	105.1 (2)	O2^iv^—Cs—O5^iii^	126.15 (10)
O3—As1—O4	106.8 (2)	O4^ii^—Cs—O5^iii^	136.61 (10)
O5—As2—O6	115.3 (2)	O5^iii^—Cr—O7	91.2 (2)
O5—As2—O7	115.6 (2)	O5^iii^—Cr—O1^v^	172.37 (19)
O6—As2—O7	105.5 (2)	O7—Cr—O1^v^	89.22 (18)
O5—As2—O4	107.1 (2)	O5^iii^—Cr—O3	92.97 (19)
O6—As2—O4	105.98 (19)	O7—Cr—O3	90.21 (17)
O7—As2—O4	106.69 (19)	O1^v^—Cr—O3	94.65 (19)
O7—Cs—O3^i^	152.96 (12)	O5^iii^—Cr—O2^vi^	89.8 (2)
O7—Cs—O6	51.76 (11)	O7—Cr—O2^vi^	173.72 (17)
O3^i^—Cs—O6	127.61 (10)	O1^v^—Cr—O2^vi^	88.99 (19)
O7—Cs—O6^ii^	100.13 (11)	O3—Cr—O2^vi^	95.94 (17)
O3^i^—Cs—O6^ii^	106.10 (11)	O5^iii^—Cr—O6^vii^	86.15 (18)
O6—Cs—O6^ii^	78.42 (11)	O7—Cr—O6^vii^	82.96 (18)
O7—Cs—O2^i^	124.64 (11)	O1^v^—Cr—O6^vii^	86.33 (18)
O3^i^—Cs—O2^i^	51.93 (11)	O3—Cr—O6^vii^	173.08 (17)
O6—Cs—O2^i^	172.88 (11)	O2^vi^—Cr—O6^vii^	90.92 (17)
O6^ii^—Cs—O2^i^	108.67 (11)	As1—O1—Cr^iii^	154.6 (3)
O7—Cs—O7^iii^	89.34 (11)	As1—O1—Cs^ii^	100.29 (19)
O3^i^—Cs—O7^iii^	112.83 (12)	Cr^iii^—O1—Cs^ii^	95.14 (16)
O6—Cs—O7^iii^	108.42 (11)	As1—O2—Cr^viii^	139.0 (2)
O6^ii^—Cs—O7^iii^	48.86 (11)	As1—O2—Cs^ix^	96.55 (17)
O2^i^—Cs—O7^iii^	76.75 (11)	Cr^viii^—O2—Cs^ix^	117.92 (17)
O7—Cs—O1^ii^	102.27 (11)	As1—O2—Cs^x^	109.71 (19)
O3^i^—Cs—O1^ii^	80.54 (11)	Cr^viii^—O2—Cs^x^	94.09 (15)
O6—Cs—O1^ii^	50.85 (11)	Cs^ix^—O2—Cs^x^	87.81 (10)
O6^ii^—Cs—O1^ii^	71.11 (11)	As1—O3—Cr	126.1 (2)
O2^i^—Cs—O1^ii^	131.26 (11)	As1—O3—Cs^ix^	100.80 (17)
O7^iii^—Cs—O1^ii^	119.98 (11)	Cr—O3—Cs^ix^	128.28 (18)
O7—Cs—O2^iv^	78.78 (11)	As2—O4—As1	118.7 (2)
O3^i^—Cs—O2^iv^	82.70 (11)	As2—O4—Cs^ii^	97.91 (16)
O6—Cs—O2^iv^	53.39 (11)	As1—O4—Cs^ii^	95.06 (16)
O6^ii^—Cs—O2^iv^	119.42 (10)	As2—O5—Cr^v^	162.6 (3)
O2^i^—Cs—O2^iv^	121.35 (13)	As2—O5—Cs^v^	85.37 (19)
O7^iii^—Cs—O2^iv^	161.82 (10)	Cr^v^—O5—Cs^v^	99.46 (18)
O1^ii^—Cs—O2^iv^	50.97 (11)	As2—O6—Cr^xi^	138.4 (2)
O7—Cs—O4^ii^	140.21 (11)	As2—O6—Cs	98.02 (17)
O3^i^—Cs—O4^ii^	60.20 (10)	Cr^xi^—O6—Cs	98.31 (15)
O6—Cs—O4^ii^	92.45 (11)	As2—O6—Cs^ii^	106.32 (18)
O6^ii^—Cs—O4^ii^	49.76 (10)	Cr^xi^—O6—Cs^ii^	107.45 (16)
O2^i^—Cs—O4^ii^	92.76 (11)	Cs—O6—Cs^ii^	101.58 (11)
O7^iii^—Cs—O4^ii^	86.49 (10)	As2—O7—Cr	134.3 (2)
O1^ii^—Cs—O4^ii^	48.42 (10)	As2—O7—Cs	104.25 (18)
O2^iv^—Cs—O4^ii^	93.83 (10)	Cr—O7—Cs	115.48 (17)
O7—Cs—O5^iii^	51.51 (11)	As2—O7—Cs^v^	91.67 (16)
O3^i^—Cs—O5^iii^	132.61 (11)	Cr—O7—Cs^v^	107.45 (17)
O6—Cs—O5^iii^	98.57 (11)	Cs—O7—Cs^v^	92.57 (11)

Symmetry codes: (i) *x*−1, −*y*+1/2, *z*−1/2; (ii) −*x*+1, −*y*, −*z*+1; (iii) *x*, −*y*+1/2, *z*−1/2; (iv) *x*−1, *y*, *z*; (v) *x*, −*y*+1/2, *z*+1/2; (vi) −*x*+2, *y*+1/2, −*z*+3/2; (vii) −*x*+1, *y*+1/2, −*z*+3/2; (viii) −*x*+2, *y*−1/2, −*z*+3/2; (ix) *x*+1, −*y*+1/2, *z*+1/2; (x) *x*+1, *y*, *z*; (xi) −*x*+1, *y*−1/2, −*z*+3/2.
